# COVID-19 Impacts on Mental Health Care in Veterans Health Administration Community Living Centers

**DOI:** 10.1093/geroni/igab046.2701

**Published:** 2021-12-17

**Authors:** Jenefer Jedele, Cameron Griffin, Kim Curyto, Michele Karel

**Affiliations:** 1 Veterans Health Administration, Ann Arbor, Michigan, United States; 2 VA, Ann Arbor, Michigan, United States; 3 VA Western New York Healthcare System, Batavia, New York, United States; 4 Veterans Health Administration, Washington, District of Columbia, United States

## Abstract

COVID-19 forced VHA Community Living Centers (CLC) to adjust how mental health (MH) care is provided. Beginning March 2020, admissions and staff entering CLC space were restricted in response to the pandemic. Some care shifted from in-person to virtual. Veterans were more isolated due to visitor restrictions and cancellation of communal activities. Pre-COVID, CLC teams cared for an already complex population – 80% of residents had a MH diagnosis (24% with serious mental illness). Changing resident composition and increased isolation may intensify challenges in providing MH care. Using VHA administrative data, we assess the impact of the changing CLC environment during the pandemic by comparing monthly average rates of MH diagnoses and provision of MH care and as-needed psychotropics to CLC residents pre-COVID (Oct 2019 – Feb 2020) to the COVID period (Mar 2020 – Feb 2021). CLCs experienced a 26% decline in the monthly resident census. However, the monthly percentage of residents with a serious mental illness increased 13%. Pre-COVID, virtual MH encounters were received by 2% of residents; 35% received an in-person MH encounter. During COVID, 8% received a virtual MH encounter and 33% received in-person. As-needed antipsychotics remained unchanged, while as-needed benzodiazepine prescriptions decreased 15%. Despite increased MH concerns, CLC teams did not appear to respond with increased pharmacological interventions. Rather, teams seem to have maintained clinical service connection for those with MH concerns. Documenting successful approaches for addressing MH needs during this challenging time will be instructive for future care during times of crisis.

